# Oxidases as Oxygen Scavengers in Hypoxic Conditions: A Kinetic Model

**DOI:** 10.3390/molecules28135216

**Published:** 2023-07-05

**Authors:** Paolo Bazzoli, Stefania Iametti, Dimitrios Fessas, Francesco Bonomi, Alberto Schiraldi

**Affiliations:** Department of Food Environmental and Nutritional Sciences (DeFENS), University of Milan, Via Celoria 2, 20133 Milano, Italystefania.iametti@unimi.it (S.I.); dimitrios.fessas@unimi.it (D.F.); francesco.bonomi@unimi.it (F.B.)

**Keywords:** oxygen scavenging, glucose oxidase, laccase, hypoxic conditions, kinetic model, fluorescent probe, stopped-flow spectrophotometry

## Abstract

A simple kinetic model allowed for the description of the observed decay of the oxygen content in hypoxic aqueous samples with and without headspace, in the presence of glucose oxidase (Glucox) or laccase and their substrates (glucose for Glucox and ABTS for Laccase). The experimental tests involved both the direct measurement of the oxygen content with a fluorescence-based probe and the indirect stopped-flow spectroscopic detection of colored compounds generated from suitable chromogenic reagents. The complete depletion of dissolved oxygen occurred in the no-headspace samples, whereas some residual oxygen remained in a steady state in the samples with headspace. Simple pseudo-first-order kinetics was adequate to describe the behavior of the system, as long as oxygen was the rate-limiting compound, i.e., in the presence of excess substrates. The values of the kinetic constants drawn from best-fit routines of the data from both experimental approaches were quite comparable. The oxygen residues in the samples with headspace seemed related to the low solubility of O_2_ in the aqueous phase, especially if compared with the large amount of oxygen in the headspace. The extent of such residue decreased by increasing the concentration of the enzyme. The kinetic model proposed in this paper can be of help in assembling suitable sensors to be used for food safety and quality control.

## 1. Introduction

Residual oxygen can be seriously detrimental to the shelf life of many perishable products, such as food and pharmaceutical preparations, since it is responsible for enzymatic and non-enzymatic “browning”, even under hypoxic conditions [1,2,3]. These occur in packages inflated with inert atmosphere, which can contain residual oxygen or allow some oxygen to infuse from the exterior [4,5,6,7,8].

Oxidases can play the role of oxygen scavengers [3,9,10,11,12], either when placed in separate oxygen-permeable bags within the package, or when added to the inner layer of the packaging material itself. The flavoenzyme glucose oxidase (Glucox), as a free or immobilized enzyme, has been used in many applications in food packaging [7]. In particular, it was shown that the Glucox immobilized on the film’s surface shows tenfold higher activity than the enzyme present within the polymer matrix, although its capacity for total oxygen removal was lower than the enzyme immobilized on the film’s surface [7]. Glucox has found broad and satisfactory uses in preserving perishable products, in particular when coupled with enzymes capable of decomposing its hydrogen peroxide byproduct (such as catalase), or using hydrogen peroxide as a reagent for further oxidation steps (such as peroxidases) [4,13,14,15]. The presence of either of these “secondary” enzymes also minimizes the inhibiting effects of H_2_O_2_ on the activity of Glucox [16]. The Glucox/HRP and Glucox/catalase combinations are the subjects of some papers ([6] and therein quoted works), although, to our knowledge, no direct comparison has been reported. These issues are indeed interesting for food technology, but out of the scope of the present work, which addresses only the simplified kinetic approach that describes oxygen scavenging in a given system with oxidases added. Laccase, a copper-based enzyme, has found similar uses, including a broad number of applications in an immobilized state [12,17].

In the case of Glucox, some drawbacks are associated with the formation of gluconic acid, which lowers the pH of the product. However, in the case of alkaline foods—such as egg white—this effect seems desirable, as it lowers the rates of non-enzymatic glycation and non-enzymatic browning reactions. In the case of laccase, adverse effects may be due to the many metal chelators present in foods (competing for copper ligands in the enzyme active site and leading to loss of activity) or to supplementation with metal ions that may compete with the active site copper and may displace it.

When trying to address, in a non-phenomenological way, the use of oxidases as components of any oxygen scavenging systems, one major issue to face is represented by the balance between oxygen consumption by the enzyme/co-substrate couple and the partial permeability of wraps, containers, lids, etc., to external oxygen [6]. Analysis of the system is further complicated by any residual oxygen in the headspace, and by the associated requirement for gas transfer, as well as by the hypoxic conditions often associated with the use of modified atmospheres in real-life food packaging.

A quantitative assessment of these issues requires a suitable description of the relevant kinetics. The literature reports some very detailed analyses of the redox cycles of the two enzymes [18,19,20,21,22], dissecting the function and role of various active and non-active species, as well as a number of intermediate products, that come into play according to the sequence of their formation, their concentration, and their stability. Thus, any kinetic model of the overall redox cycle for each enzyme implies a number of differential equations to account for the many steps, each with its peculiar rate [16,18,19,20,21]. Obviously, on top of this, factors such as enzyme concentration and activity come into play, along with physical and chemical reaction parameters, such as temperature, pH, water activity, and ionic strength. In the particular case of oxidases at large, the overall scenario includes the possible concomitant occurrence of non-enzymatic reactions that may involve just some reaction intermediates.

However, from a practical standpoint, the detailed kinetic model required for a comprehensive understanding of the relevant biochemistry in these enzymatic cycles is not necessary, given that the predetermined environmental conditions associated with the typical food industry application may allow one to disregard many variables. For example, under given physical and chemical conditions, the supply of substrates (glucose/oxygen in the case of Glucox, or phenolics/oxygen in the case of laccase) can become rate-limiting with respect to the whole redox cycle, in particular when assuming that all the other involved compounds are present in large excess.

The experimental approach described in this paper concerns the direct determination of the oxygen content in a liquid matrix with a fluorescent probe (Oxysense 101 (Dallas, TX, USA), see below), and the indirect tests based on fast kinetic spectrophotometry at a selected wavelength to monitor the evolution of colored compounds formed in the presence of excess glucose and specific substrates. In the case of Glucox, this approach requires the use of horseradish peroxidase (HRP), which allows the oxidation of guaiacol (in excess) to form a colored quinone. Both approaches lead to quantitatively comparable results that support a simplified kinetic model that can describe the scavenging of molecular oxygen from aqueous solutions with added glucose oxidase (Glucox) and laccase under conditions of practical relevance.

These are (i) the presence/absence of a headspace; (ii) varying oxygen concentrations, covering the whole range from normal to hypoxic conditions; (iii) or the presence of a large excess of the appropriate co-substrate. Such conditions pertain to industrially relevant situations and are suitable to analyze the broadest possible range of oxygen concentration in the system. These conditions are also of interest for the assembly/setup and control/management of active packaging materials/structures that can host enzyme-based oxygen sensors, either adherent to the packaging surface or embedded in any of the coating layers.

## 2. Results and Discussion

**Figure 1:**
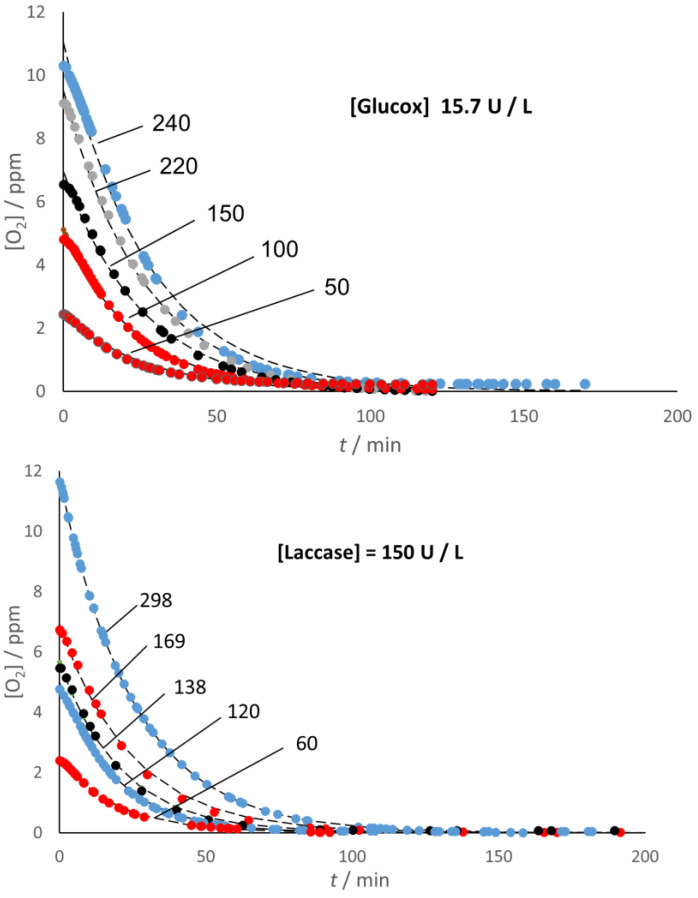
Decay of the O_2_ content in solutions without headspace. Lettering stands for *p*(O_2_)/hPa in the gas mixture used to prepare the solutions. The fitting curves (dotted lines) correspond to exponential functions (see text).

The use of the Oxysense 101 device allowed a direct way to monitor the decrease of O_2_ content with both enzymes and under both conditions (presence/absence of headspace) used in this study. According to the experimental evidence, Figure 1 indicates that both enzymes were acting as effective oxygen scavengers even under hypoxic conditions, leaving no residual O_2_ in the system, no matter the initial O_2_ content, when the sample had no headspace. As explained in the section Section 3, the starting oxygen concentration, [O_2_]*_i_*, was calculated from the value of the partial pressure of oxygen, *p*(O_2_), in the (Ar/O_2_) gas mixture used to condition the environment where the aqueous solution was prepared.

Figure 1 also provides graphical evidence that simple exponential decay can fit very well the kinetics of the O_2_ depletion in the no-headspace samples, regardless of their initial O_2_ content. The fitting function used was:

(1) $$\left[O_{2}\right]={\left[O_{2}\right]}_{i}\exp\left(-k_{1}t\right)$$

where *k*_1_ = *k*_1G_ = 0.039 min^−1^, and *k*_1_ = *k*_1L_ = 0.046 min^−1^ for Glucox and Laccase, respectively, in fair agreement with the values calculated (as the ratio V_max_/K_M_ of Michaelis–Menten parameters) from the literature data [21,22].

This interpretation implies that the O_2_ depletion is—at least under hypoxic conditions and in the presence of excess co-substrate (glucose and ABTS for Glucox and laccase, respectively)—the rate-limiting step in the overall cycle of both enzymes.

These results are essentially identical to those obtained from stopped-flow spectrophotometric tests where absorbance was the detected variable.

**Figure 2:**
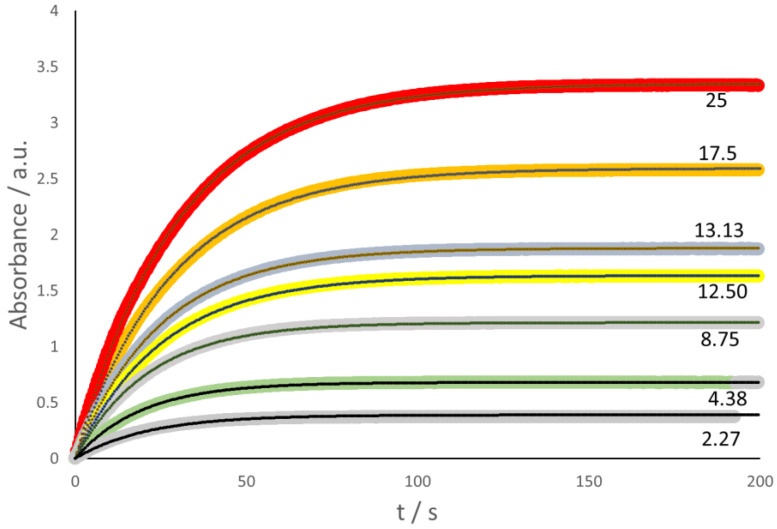
The trend of the absorbance at 436 nm wavelength of the chromophore related to ABTS-ox. The continuous black curves correspond to the assumed kinetic scheme. Each curve is accompanied by the value of the *p*(O_2_) (hPa units) in the preparation of the solutions. The laccase concentration is 10.51 U/mL.

In the case of laccase, the colored compound is ABTS-ox, formed during the reduction of laccase-ox. In other words, the spectrophotometric detection of the absorbance (*Abs*) related to ABTS-ox at 436 nm wavelength (ε = 29,300 M^−1^ cm^−1^ [20], see Figure 2) is indeed equivalent to the direct Oxysense test. The relevant kinetic scheme, therefore, reflects a simple exponential growth, and thus corresponds to the same kinetic model that describes the Oxysense data (see above), namely,

*Abs* = *Abs*_(*t*=0)_ [1 − exp(−*k*_1L_ *t*)] (2)

**Figure 3:**
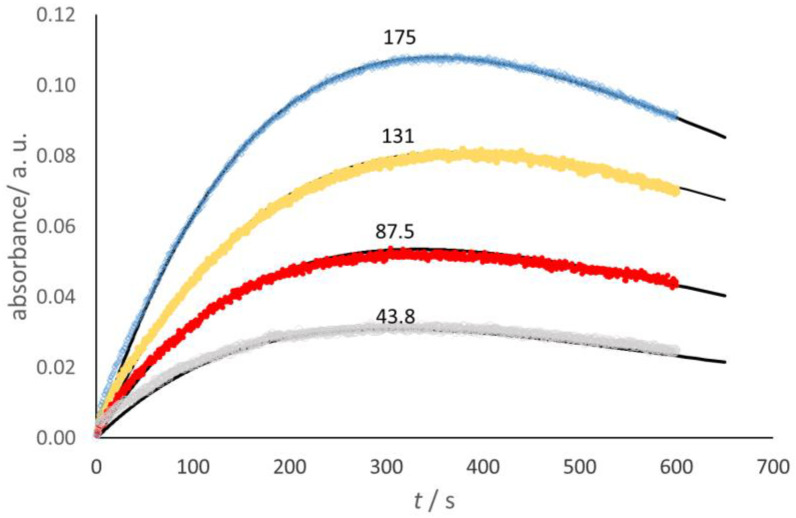
The trend of the specific absorbance at 436 nm goes through a maximum because of the loss of the colored compound (quinone) formed by oxidation of the guaiacol. The continuous black curves correspond to the assumed kinetic scheme of two successive steps (see below). [Glucox] = 131 U L^−1^; [HRP] = 18.82 U mL^−1^. Each curve is accompanied by the value of the *p*(O_2_) (hPa units) in the preparation of the solutions.

In the case of Glucox, the stopped-flow spectrophotometric investigation made use of the coupled process, where guaiacol was oxidized by the H_2_O_2_ produced in the Glucox cycle, thanks to the presence of horseradish peroxidase (HRP), to form a colored quinone that showed a broad-shouldered absorbance maximum in the 420–470 nm range [23]. The absorbance at 436 nm [24] was selected for the present work (Figure 3).

The dome-shape trends shown in Figure 3 reveal that, as expected, the quinone decays to form other products [23], which were detected in the present work with HPLC tests. Taking into account the stoichiometry, namely, 1 mole of O_2_ yields 4 moles of quinone, a simplified kinetic model allows the fit of the absorbance trend, namely,

(3) $$O_{2}\overset{k_{2}}{\to }Quinone(colour)\overset{k_{3}}{\to }\sum Pi$$

where *k*_2_ = 0.25 *k*_1G_. This corresponds to the known equation for two consecutive steps,

(4) $$\left[Q\right]=\left(\frac{k_{3}{\left[O_{2}\right]}_{i}}{k_{4}-k_{3}}\right)\left[exp(-k_{2}t)-exp(-k_{3}t)\right]$$

where [*Q*] is the quinone concentration.

**Figure 4:**
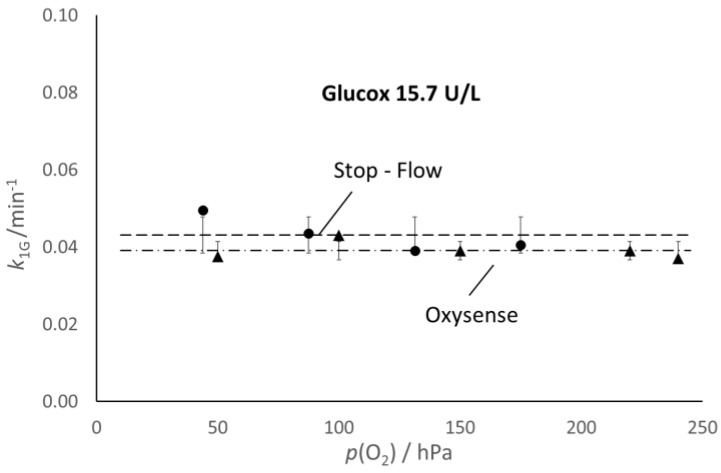

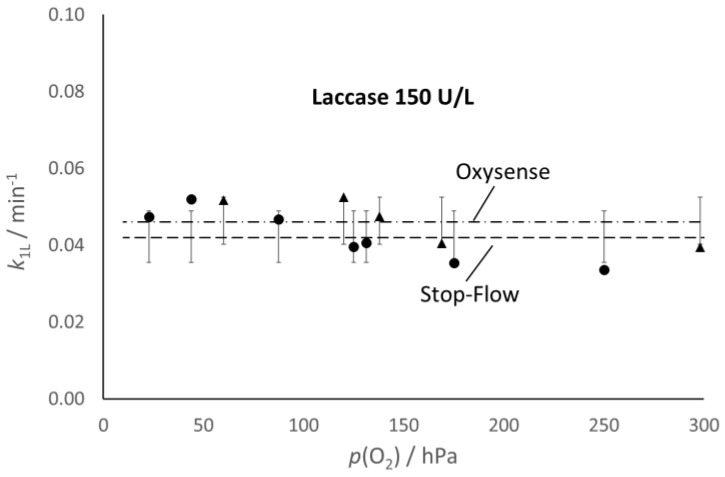
Comparison of the kinetic constant determined with the Oxysense 101 device (triangles and dot-dashed line) and the stopped-flow spectrophotometric approach (full dots and dashed line). Bars correspond to the standard deviation.

From the *k*_2_ values determined through the fitting routine, one can calculate the values of *k*_1G_ reported in Figure 4, which allows the comparison of the values of the kinetic constants, k_1G_ and k_1L_, determined with the Oxysense (0.039 and 0.046 min^−1^, respectively) and the spectrophotometric approach (0.043 and 0.042 min^−1^, respectively).

The presence of a headspace hosting some oxygen substantially modified the picture. For these experiments, the direct monitoring of the oxygen content was the only approach used, since the stopped-flow spectrophotometric approach was not possible.

**Figure 5:**
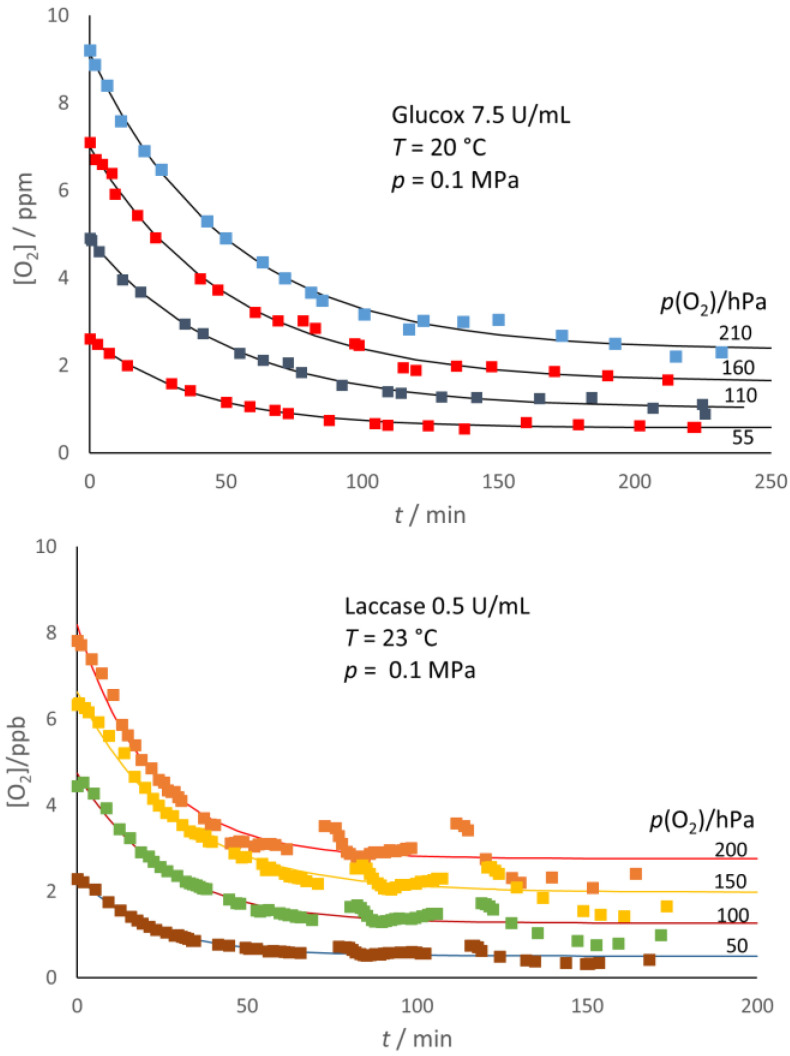
Oxygen scavenging in the liquid phase by the oxidases in the presence of headspace with various initial *p*(O_2_) in the headspace. The fitting lines correspond to a simple kinetic model (see text).

**Figure 6:**
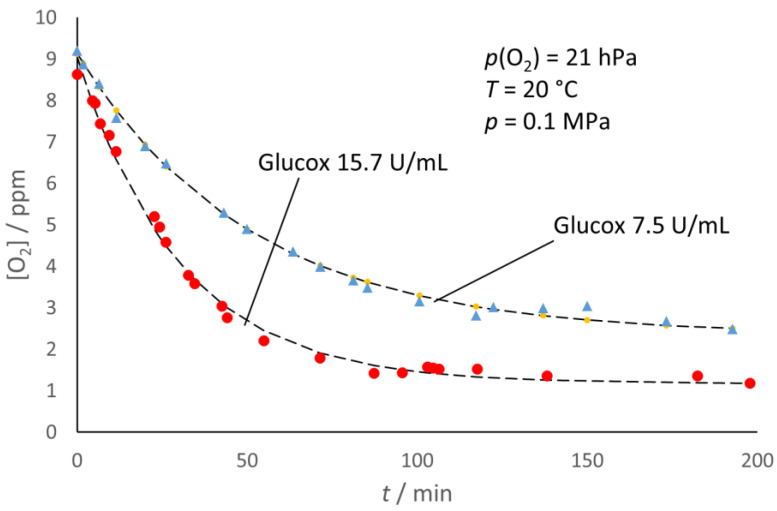
Effects of the enzyme concentration on the time course of O_2_ depletion from the liquid phase in the presence of headspace. For each experimental condition, lines correspond to the simplified kinetic model (see text).

As shown in Figure 5, the decrease in oxygen content after the enzyme addition was not completed with either enzyme and, in the end, reached a steady concentration, [O_2_]*_e_*, which appeared lower than the initial oxygen content, [O_2_]*_i_*, of the sample. In addition, as exemplified in Figure 6 for glucose oxidase, the [O_2_]*_e_* decreased with the increasing enzyme concentration.

The starting condition (namely, before the injection of the enzyme) reflects a true solubility equilibrium, [O_2_]*_i_* = K_H_ *p_i_*(O_2_), with K_H_ being the Henry constant. The depletion of oxygen from the liquid phase would trigger the transfer of oxygen from the headspace to the liquid phase to re-establish equilibrium. Were the oxygen transfer too slow with respect to the enzymatic scavenging, one would observe a complete oxygen depletion from the liquid phase. However, what actually occurs is competition between the processes, with the scavenging of the oxygen supply from the headspace initially ahead. However, as the oxygen concentration in the liquid phase, [O_2_], decreases, the scavenging rate also decreases, since it is proportional to [O_2_]. The system finally reaches a steady condition (for the duration of the experiment); namely, the rate of oxygen supply from the headspace counterbalances the scavenging rate: the oxygen concentration in the liquid phase attains a steady value, [O_2_]*_e_*. The experimental evidence presented in Figure 5 and Figure 6 allowed a simple kinetic interpretation of the observed trends.

(5) $$O_{2}\left(G\right){\overset{k_{tr}}{\to }O}_{2}\left(L\right)\overset{k_{1}}{\to }Products$$

(6) $$\frac{d\left[O_{2}\right]}{dt}=k_{tr}p\left(O_{2}\right)-k_{1}\left[O_{2}\right]$$

where *k*_tr_ and *k*_1_ are the kinetic constants, with *k*_1_ being proportional to the enzyme concentration.

In the final steady condition, (*d*[O_2_]/*dt*) = 0, which means that

*p*_e_(O_2_) = (*k*_1_/*k*_tr_) [O_2_]*_e_*. (7)

As for the starting condition, one may use the equilibrium constant, namely,

*p*_i_(O_2_) = (1/*K*_H_) [O_2_]*_i_.* (8)

The steady condition is long-lasting because of two main reasons:

- the solubility of oxygen is very low (see Table 1), and
- the amount of oxygen in the headspace (18 mL, about 40 μmoles for *p*(O_2_) = 50 hPa) is much larger than in the liquid phase (4 mL, about 0.05 μmoles). This also implies a very small variation in *p*(O_2_) in the headspace.

For a given enzyme concentration, the ratio between the initial and end values of the oxygen content in the liquid phase therefore is

(9) $$\frac{{\left[O_{2}\right]}_{i}}{{\left[O_{2}\right]}_{e}}=\frac{K_{H}}{\left(\frac{k_{tr}}{k_{1}}\right)}\frac{p_{i}(O_{2})}{p_{e}(O_{2})}\sim K_{H}\left(\frac{k_{1}}{k_{tr}}\right)$$

while the difference ([O_2_]*_i_* − [O_2_]*_e_*) is

(10) $$\left({\left[O_{2}\right]}_{i}-{\left[O_{2}\right]}_{e}\right)=\Delta \left[O_{2}\right]={\left[O_{2}\right]}_{i}\left(\frac{k_{1}-k_{tr}}{K_{H}k_{1}}\right)=p_{i}\left(O_{2}\right)\left(\frac{k_{1}-k_{tr}}{k_{1}}\right)$$

**Figure 7:**
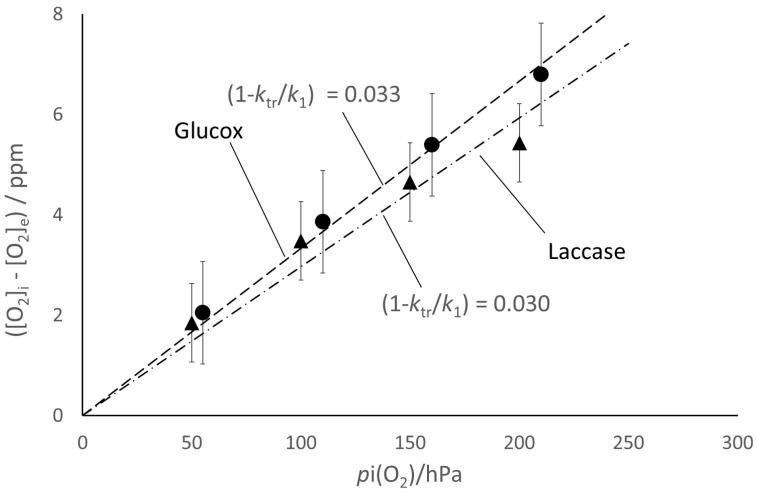
The extent of the decay of oxygen content is directly proportional to the initial partial pressure of oxygen in the headspace, *p_i_*(O_2_), namely, the one in the gas mixture used to prepare the solutions. Bars correspond to the standard error.

The corresponding fit of the experimental data (Figure 5 and Figure 6) seems satisfactory for both enzymes, in spite of the underlying naïve kinetic model that is much simpler than the literature reports [15,16,17,18,19]. The model also accounts for the observed larger extent of the [O_2_] decay for the larger enzyme concentration (Figure 4) and for the correlation between ([O_2_]*_i_* − [O_2_]*_e_*) and *p*_i_(O_2_) (Figure 7). From the slope of the straight-line fits reported in Figure 7, one can estimate (*k*_tr_/*k*_1_) ≈ 0.97 for both enzymes, which reflects the balanced competition between the transfer and scavenging rates.

The residue of oxygen in real packages that allow some headspace can have adverse consequences for the quality and/or safety of very perishable products. The results of the kinetic approach presented above allow the definition of some guidelines for the packaging practice. Relatively stable products can tolerate some residual oxygen for some hours (or days); they do not require vacuum or inert atmosphere packaging in the presence of an oxygen scavenger, such as those considered in this work. The residual oxygen actually does not persist indefinitely, as the apparent steady level does not correspond to a true equilibrium condition, but slowly decays because of the scavenging action of the enzyme. A larger concentration of the enzyme and/or a reduction of the *p*(O_2_) in the headspace will accelerate the oxygen depletion, as suggested by the data reported in Figure 6 and Figure 7. Very sensitive products would, instead, require vacuum and/or inert atmosphere packaging. In such cases, the enzyme scavenging action would concern just the oxygen traces coming from the production lines and/or infused from the exterior through the packaging, thereby prolonging the overall shelf life of the product.

## 3. Materials and Methods

### 3.1. Chemicals and Enzymes

The stock aqueous glucose (1 M) was prepared from anhydrous D-(+) glucose. The solution of glucose oxidase (E.C. 1.1.3.4), type X-S, from *Aspergillus niger*, was purchased (Sigma G 7141, St. Louis, MO, USA, 157 U/mg); one oxidase unit corresponds to the formation of 1.0 μmol/min of H_2_O_2_ (or gluconolactone) at pH 5.1 and 35 °C. The coupled oxidation of guaiacol in the presence of horseradish peroxidase (E.C. 1.11.1.7; Sigma P 6140, type X, 291 U/mg) produced a colored quinone (maximum absorbance at 436 nm) allowed the monitoring of the formation of H_2_O_2_. The producer indicates that one peroxidase unit transforms 1 mg of purpurogallin to pyrogallol in 20 s at pH = 6.0 and 20 °C. The assay solution for the glucose oxidase activity measurements had the following composition: 170 volumes of 0.053 mg/mL guaiacol (Fluka 50880, Waltham, MA, USA) in 0.1 M phosphate buffer, at pH 7.2; 30 volumes of aqueous 1 M glucose (Fluka 49138), and 1 volume of a 1 mg/mL peroxidase in 0.1 M phosphate buffer, at pH = 6.0, containing 3.2 M (NH_4_)_2_SO_4_.

Laccase from *Agaricus bisporus* (E.C. 1.10.3.2; Fluka 40452; lyophilized powder with a specific activity of 6.3 U/mg) was used as a 25 mg/mL solution in 50 mM MES buffer, at pH 5.0. One laccase unit is defined as converting 1 μmol/min of catechol into the corresponding quinone at pH 6.0 and 25 °C. The spectrophotometric investigations of the laccase activity concerned the absorbance at 420 nm wavelength by the oxidized ABTS (2,2′-azino-bis(3-ethybenz-thiazoline-6 sulfonic acid) diammonium salt (Sigma A1888), as a substrate, in 0.05 M MES buffer at pH 5.0.

### 3.2. Spectrophotometric Measurements

A stopped-flow mixer (Bio Logic MPS-52, Seyssinet-Pariset, France) connected to a circular dichroism spectrometer (JASCO J810, Cremella, LC, Italy) was used for investigating the reaction kinetics. In this particular system, a stepping motor drove gas-tight syringes through a mixing chamber and a cylindrical microcuvette (2 mm optical path, total capacity 0.08 mL), placed in a four-window observation head that allows the simultaneous detection of fluorescence and transmittance/absorbance. Stopped-flow absorbance measurements were carried out from 0 to 10 min at a given wavelength (436 nm for both the Glucox and laccase tests) with a bandwidth of 4 nm. Dedicated software (Bio Logic MPS-52) was used for the data analysis.

### 3.3. Measuring the Oxygen Removal

The tests of enzymatic activity concerned two main situations: (A) a two-phase system, namely, liquid and gas (large headspace); (B) a single liquid phase with a given oxygen initial concentration (no headspace).

(A) Measurements in the presence of a headspace

**Figure 8:**
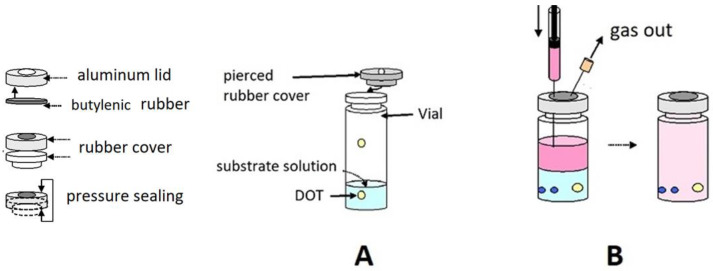
Physical features and arrangement of the vials for measurements carried out in the presence of a large headspace (**A**) or in the absence of a headspace (**B**). In both cases, the yellow dots represent the solid-state oxygen sensors (see below) glued to the inner wall of the vial. The blue spheres in panel (**B**) represent glass beads that allow thorough mixing of the reagents in the absence of a headspace.

An oxygen sensor (DOT, see below) was glued with a silicon adhesive (RV 118) to the internal wall of a standard 22 mL vial, fitted with a gas-tight, perforable rubber stopper, and sealed with an aluminum lid and a butylenic rubber frame (Figure 8A). The vials were purged with at least three vacuum/Ar cycles through a standard vacuum line, before being filled with Ar/O_2_ mixtures (approximately 5–10–15–20% oxygen, *v*/*v*) prepared in a mixer (Map Mix PBI Dansensor) at an overall constant pressure of 0.1 MPa. The actual composition of each gas mixture was checked with a gas chromatograph (Hewlett Packard HP 5870 SERIES II), equipped with a thermo-conductivity sensor and a CTR I stainless steel column (2 m × 6 mm, Alltech Italia Srl, Casalecchio di Reno, Italy).

A standard vacuum line was used to prepare the anaerobic reaction mixtures under Ar. gas-tight syringes, which allowed the transfer of a 4 mL solution of a given composition to each vial and achieve equilibration with the adjusted gas phase. The corresponding oxygen concentration in the liquid phase was checked to compare the response of the Oxysense 101 probe (in the absence of enzyme) and the oxygen solubility at various *T* (Table 1).

The vials underwent the OxySense 101 tests immediately after the anaerobic addition of the required enzyme (in negligible volumes). The samples for the stopped-flow tests were the same solutions contained in the vials, withdrawn with gas-tight syringes (replacing the withdrawn volume with an identical volume of Ar at 0.1 MPa) and directly injected in the stopped-flow apparatus.

(B) No-headspace measurements

In this case, each vial was completely filled with appropriate mixtures of substrate solutions prepared either anaerobically under Ar or equilibrated with air at atmospheric pressure, that is, at *p*(O_2_) = 210 hPa (Figure 8B) at room temperature. Cooling the air-exposed mixtures allowed the attainment of slightly higher oxygen contents.

### 3.4. Principle and Operation of the Oxysense 101

**Figure 9:**
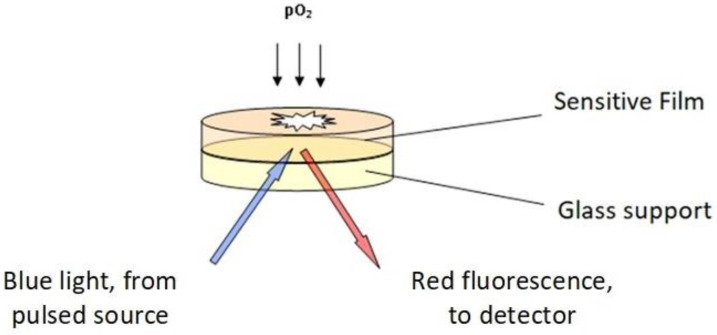
Schematic picture of the Oxysense 101 device.

Figure 9 reports a scheme of the sensing Oxysense 101 device that detects the effects of oxygen on the fluorescence of a ruthenium/phenanthroline complex, which is one of several specific biosensors [25,26,27]. The fluorescence decay obeys the law *I*/*I*_0_ = exp(−t/τ), where τ is the decay time and *I* stands for the intensity of the emitted fluorescence. The decay time is related to the partial pressure of oxygen through the Stern–Volmer equation, τ_0_/τ = 1 + K_SV_ *p*(O_2_), often written in the form (1/τ = A *p*(O_2_) + B) [27]. Since the parameters A and B in the last equation depend on the temperature and on minor modifications of the composition among individual lots of the sensitive film, a preliminary calibration of each lot of film at various temperatures is necessary. Noteworthily, the sensitive film used in this device is stable in the temperature range 0–90 °C, and over the 2–11 pH range.

In the used vials, illustrated in Figure 8, a hydrophobic polymeric film (100 μm thick) hosting the reactive mixture (4,7 biphenyl 1,10 phenanthroline and ruthenium chloride) is glued onto a glass disc (5 mm diameter and 0.17 mm thick). An external pulsed LED acts as the blue-light source. Using a pulsed light allows computer-assisted measurement of the decay rates of the red fluorescence emitted by the ruthenium complex in the film (1 to 5 μs), in the presence and absence of oxygen, respectively). Dedicated software then converts the fluorescence decay rates into the oxygen content in the gas phase, *p*(O_2_), in the % of the 1000 hPa total pressure, whereas the oxygen content of the liquid phase (in μg/mL, ppm) was calculated from tabulated oxygen solubility data (Table 1).

## 4. Conclusions

The fluorescent probe and spectrophotometric tests allowed the monitoring of the decay of the oxygen content of the hypoxic samples in the presence of glucose oxidase and laccase. The collected data showed that, in hypoxic conditions and for a given enzyme concentration, the oxygen concentration was the rate-limiting factor of the redox cycles. This evidence justified the use of simple pseudo-first-order kinetics to describe the observed trends. Both enzymes seemed to be efficient oxygen scavengers, both in the presence or in the absence of headspace.

The whole oxygen content of the liquid phase was exhausted in the samples with no headspace. In the presence of some headspace, the oxygen content of the liquid phase attained a residual value that appeared quite steady (for the duration of the experiment). This, presumably, was a consequence of the balance between the transfer of oxygen from the headspace and the scavenging action of the enzyme. In view of the damaging effects of such residues in packaged food, the experimental evidence showing that the residues of oxygen were smaller for larger concentrations of the enzyme could be of some interest.

The simplified kinetic model of the oxygen scavenging activity of these oxidases in hypoxic conditions can be of help not only for monitoring practices of food and pharmaceutical industries, but also for the future developments of biosensors.

## Figures and Tables

**Table 1:** Solubility of oxygen in water at various temperatures. Adapted from Orbisphere table accompanying the 101 device.

*T* (°C)	0	5	10	15	20	25	30	35	40
[O_2_] (ppm)	14.60	12.73	11.26	10.07	9.07	8.21	7.53	6.92	6.38

## Data Availability

All the detailed data are available on request addressed to the corresponding author, Alberto Schiraldi, at alberto.schiraldi@unimi.it.
